# Decoupling the Effects of Temperature, Strain, and Refractive Index in Long-Period Fiber Grating Used for Epoxy Resin Cure Monitoring

**DOI:** 10.3390/s25030786

**Published:** 2025-01-28

**Authors:** Oleg V. Ivanov, Kaushal Bhavsar, James M. Gilbert

**Affiliations:** School of Engineering, University of Hull, Hull HU6 7RX, UK; k.bhavsar@hull.ac.uk (K.B.); j.m.gilbert@hull.ac.uk (J.M.G.)

**Keywords:** long-period fiber grating, epoxy resin, cure monitoring, refractive index sensing

## Abstract

Epoxy resins are widely used in the manufacture of composite materials for a wide range of applications. Control of the curing process is an important consideration in ensuring product quality and minimizing production times. The curing of epoxy resin is associated with temperature, strain, and refractive index changes but it is difficult to monitor these quantities individually and hence difficult to achieve accurate control of the curing process. One promising approach for monitoring these quantities is the use of long-period fiber gratings (LPFG). We analyze the spectral response of a LPFG in epoxy resins to temperature, strain, and refractive index. Wavelength shifts and dip amplitudes of cladding mode notches are monitored and are used to decouple temperature, strain, and refractive index for gratings in air, liquid, and hardened resins. The three measurands are found from wavelength shifts and dip amplitudes, employing multiplication by a weighted pseudo-inverse matrix assuming linear dependences between the spectral and external parameters. We propose a new model to describe the influence of fiber parameters and external refractive index, temperature, and strain on the spectral behavior of long-period fiber gratings in epoxy resins during hardening. The results obtained can be utilized for multiparameter cure process monitoring of epoxy resins by using long-period fiber gratings.

## 1. Introduction

Epoxy resins are widely used in the manufacturing of composite materials for various industrial applications. Composite materials are light in weight, offer high strength, and are chemically resistant. These unique properties make them ideal candidates for manufacturing various products and components in automotive, aerospace, and other large-scale civil infrastructures. Extensive use of these materials in the manufacturing of large wind turbine blades and the cost involved in the production and repair of damaged parts has created demand to improve the quality of the product [1]. The manufacture of large composite structures typically involves the infusion of liquid resin into dry glass or carbon fiber reinforcement, with resin subsequently cured to form a solid composite structure. The quality of the resulting composite materials depends on the integrity of the infusion and curing processes. To improve and optimize the epoxy curing, the manufacturing process should be continuously monitored and controlled in real time. Such a monitoring system may also give pre-alerts about defects arising during the manufacturing process which can allow operators to take measures to prevent them.

Various methods have been used to monitor the epoxy resin curing process, including acoustic, ultrasonic, and optical methods such as Raman, fluorescence, and FTIR spectroscopies; methods based on dielectric analysis of permittivity and dielectric loss factor [2,3,4]. Amongst these methods, optical fiber-based methods have gained popularity in recent years due to several inherent advantages. Optical fibers are small in size, immune to electromagnetic interference, chemically inert, and offer long-term stability; many sensors can be integrated into the same fiber with the capability to measure multiple parameters and offer ease to embed during manufacturing of composite materials without degrading their mechanical properties [5,6].

Fiber optic sensors employ interrogation methods based on measurements of intensity, wavelength, frequency, or polarization. They measure parameters such as refractive index (RI), temperature, strain, etc. Most of the existing fiber optic interrogation methods to monitor the epoxy resin curing process have used optical fiber structures such as Fabry–Pérot interferometers [7], fiber Bragg gratings (FBGs) [8], and long-period fiber gratings (LPFGs) [9]. LPFGs are of special interest due to their ability to measure the RI of external media by the interaction of the evanescent field of cladding modes with an external environment [10,11,12]. A drawback in the application of LPFGs and FBGs is their cross-sensitivity to temperature and strain [8]. On the other hand, this cross-sensitivity has allowed some researchers to discriminate between temperature and strain effects [13,14,15,16]. During the epoxy resin curing process, parameters such as RI, temperature, and strain change simultaneously, making it difficult to extract these quantities separately.

In this paper, we investigate how the RI sensitivity of LPFGs is affected by the strain and temperature variations occurring during resin hardening. We seek to decouple these parameters by measuring the spectra of LPFGs and analyzing wavelength shifts and amplitude changes of their resonance dips. The spectral response of the LPFGs to strain, temperature, and RI changes is measured in air, liquid, and hardened resin. Wavelength shifts and dip amplitudes of cladding mode notches are used as the input parameters. We present a model describing the influence of fiber and external parameters on the spectral behavior of long-period fiber gratings in epoxy resins during hardening and use a pseudoinverse of this model to disaggregate the external parameters. We discuss how the results obtained can be utilized for multiparameter cure process monitoring of epoxy resins by using long-period fiber gratings.

## 2. Long-Period Fiber Gratings for Multiparameter Sensing

LPFGs are created in an optical fiber by periodically modulating the RI of the fiber core. In order to induce this modulation, the most widely used technique employs periodic illumination of the fiber with an ultraviolet laser with typical periods in the range from 100 μm to 1 mm. Coupling between core and copropagating cladding modes creates resonant attenuation bands, which can be seen in the form of dips in the transmission spectra of LPFGs [11]. These dips are sensitive to changes in surrounding environmental parameters such as temperature, strain, RI, etc. [10].

The sensitivity of LPFGs to these parameters can be defined by measuring the change in amplitude or shift in wavelength of the resonant dips. The change in wavelength can be described by the following equation:

(1) $$∆\lambda =-\frac{∆\beta_{q}-∆\beta_{p}+2\pi s/\Lambda }{\frac{d\beta_{q}}{d\lambda }-\frac{d\beta_{p}}{d\lambda }},$$

where $∆\beta_{p}$ and $∆\beta_{q}$ are changes in the mode propagation constants caused by external environmental factors such as temperature, strain, or RI for the modes *p* and *q*; *s* is the strain value defining the change in the grating period Λ [17]. The changes to the propagation constants are small but their contribution is magnified by a small denominator containing the difference between the gradients of the two interacting modes.

The amplitudes of the LPFG dips are determined by the coupling coefficient between the core and cladding modes or interference effects if the grating is inhomogeneous. The amplitudes of the dips can also change with the RI of the external medium when it is higher than the RI of the silica fiber cladding.

When the fiber with an LPFG is heated, the RI is changed due to the thermo-optic effect and the fiber is thermally expanded. The thermal expansion coefficient of fused silica is rather small; therefore, the contribution of the thermal expansion can be neglected for silica fibers. The change in RI results in modifications of propagation constants of modes and a shift in resonance wavelengths. This shift depends on the fiber structure and the order of the cladding mode.

Compared to fiber Bragg grating, the mechanisms of strain and temperature sensitivities are more complicated because the interacting core and cladding modes propagate in the same direction and the strength of the effect depends on the difference between the properties of the two modes. In heated fiber, the refractive index is isotropically changed due to the thermo-optic effect and the fiber is isotropically deformed due to the thermal expansion. The changes in propagation constants can be found by calculating the coupling coefficients of fiber modes. There are two types of coupling coefficients: one type describes self-coupling and the other describes inter-mode coupling. The self-coupling changes the propagation constants of modes and shifts the LPFG resonances, while the inter-mode coupling transfers energy between different modes and changes the amplitudes of the transmission dips.

LPFGs fabricated in standard optical fibers exhibit temperature sensitivities in the range 30–100 pm/°C [10]. Through the choice of composition and structure of the fiber and the order of the cladding mode, it is possible to enhance the temperature sensitivity of an LPFG or, inversely, make it temperature insensitive.

When a fiber with a LPFG is axially strained, the RI is changed due to the strain-optic effect and the fiber is mechanically elongated. This results in modifications of propagation constants of modes and the period of the grating. The following factors may contribute to modified propagation constants in the presence of stress-induced deformation of the fiber: first, the dielectric permittivity of the fiber is changed due to the photoelastic effect; second, the fiber diameter is changed under strain (the core refractive index profile is compressed under the stretching deformation); third, there is coupling due to the difference between the transverse and the longitudinal components of photoelastic constants; fourth, the photoelastic coefficient is modified in the doped region of the core; and, fifth, the photoelastic effect is absent in air [17].

The resonance condition is changed, and the resonances are shifted. LPFGs fabricated in standard optical fibers exhibit strain sensitivities up to 20 pm/με [12]. It is possible to fabricate fiber LPFGs with positive or negative sensitivity to strain by appropriate choice of fiber and grating period.

When the fiber with an LPFG is immersed in a medium with an RI different from the RI of air, the propagation constant of the cladding mode is increased due to the evanescent field of the mode propagating in the vicinity of the fiber cladding. At the same time, the propagation constant of the core mode is not changed, since its evanescent field is in the cladding region. Therefore, the central wavelengths of the attenuation bands shift with the external refractive increasing up to the RI of the cladding, exhibiting weak changes in their amplitudes. When the RI of the external medium becomes higher than that of the silica cladding, the waveguiding regime is changed, and the cladding modes become radiation modes. The power that these modes radiate to free space depends on the RI difference between the cladding and the external medium, and the transmission amplitudes depend strongly on this difference. On the other hand, the central wavelengths are almost unchanged in this regime. Since the RIs of the epoxy resins we are interested in are higher than 1.45, we are going to consider only this regime of radiation modes.

In the regime with $n_{\mathrm{ex}}>n_{\mathrm{cl}}$, the guidance of light through the fiber occurs due to Fresnel reflection, and light energy is partially lost during propagation. The wave goes into the external medium, creating an outgoing wave propagating infinitely. The field amplitude of this wave oscillates in the external medium with its Poynting vector directing from the fiber. These are the radiation modes, which have continuously changing propagation constants. In a LPFG, the core mode is coupled to the continuous set of the radiation modes. A change in the external RI modifies the reflection coefficient at the cladding surface, which results in a change in the intensity of the transmitted light. This is observed as a change in the amplitude of the corresponding cladding mode resonance in the grating spectrum. In this case, the wavelength of the transmission dips does not change significantly. The amplitude of the dips increases with the RI. When the RI is high, there is good reflection at the cladding outer interface due to the Fresnel reflection and the resonances are deep. When the RI is close to the RI of silica, reflection vanishes, which results in the disappearance of the dips [9].

LPFGs can be used to monitor the curing process in epoxy resins [9,18]. The main parameter measured in this case is the RI of the external medium. However, there are other parameters such as temperature and strain that can significantly influence the transmission spectrum of the LPFG. Resin curing is an exothermic process, and a lot of heat can be produced during resin curing. Therefore, the temperature is not constant and can increase to 200 °C and return to the ambient temperature during a complete curing cycle. At the same time, strong strains are induced in the resin after curing, which is also related to the changing temperature of the resin and the chemical cross-linking which occurs. LPGFs are sensitive both to temperature and strain, and can also be influenced indirectly through the RI index of the resin, which changes under temperature and strain. Therefore, three factors contribute to the spectra of LPFG inside resin: RI, temperature, and strain. As we described above, the spectral dips may shift or change amplitude under these stimuli. The response of LPFG to these factors is defined first by the fiber and grating parameters and second by the resin parameters, such as the thermo-optic and strain-optic coefficients of both fiber and resin.

## 3. Experiment

In order to understand the behavior of LPFGs in resin, we investigate the response of a LPFG to various combinations of changes in RI, temperature, and strain in liquid and solidified resins. First, we measure the response of the LPFG itself, without resin, to changes in every single parameter and, second, the LPFG in liquid and hardened resins.

**Figure 1:**
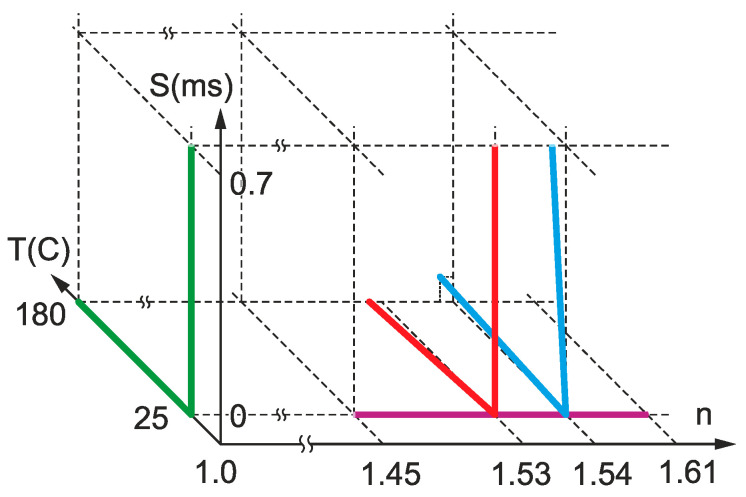
Diagram illustrating experimental studies in a 3D parameter space of RI, temperature, and strain (*n*, *T*, *S*). The green lines correspond to measurements in air, the purple line is for RI matching liquid, the red lines are for liquid resin, and the blue lines are for hardened resin.

Figure 1 shows a diagram illustrating experimental studies that we made in a 3D parameter space comprising RI, temperature, and strain. Each line corresponds to one experiment with one parameter changed continuously, the other parameters being constant or close to constant. There are 7 lines: fiber straining and heating in air (green lines), fiber straining and heating in liquid resin (red lines), fiber in RI matching liquid (purple line), and straining and heating of fiber in hardened resin (blue lines). All these measurements allow us to encompass the LPFG’s response to changing external parameters in full parameter space in different combinations.

Three of these lines are deflected from orientations strictly parallel to the coordinate axes. Therefore, heating of fiber in liquid resin changes the RI of the resin, shifting the line in the (n,T) plane to lower RIs due to the thermo-optic effect. Heating of fiber in hardened resin also expands the resin, creating strain in the fiber. Hardened resin strained with the fiber inside also reduces RI due to the strain-optic effect. It is assumed that no additional strain is induced in liquid resin.

We assume that the changes in the LPFG spectra are not very strong and that we can use an additive model without taking account of the nonlinear effects resulting from the product of changes in two or three parameters (such as ∆*n*∆*T*, *S*∆*T*, ∆*n*∆*T*, and ∆*nS*∆*T*).

**Figure 2:**
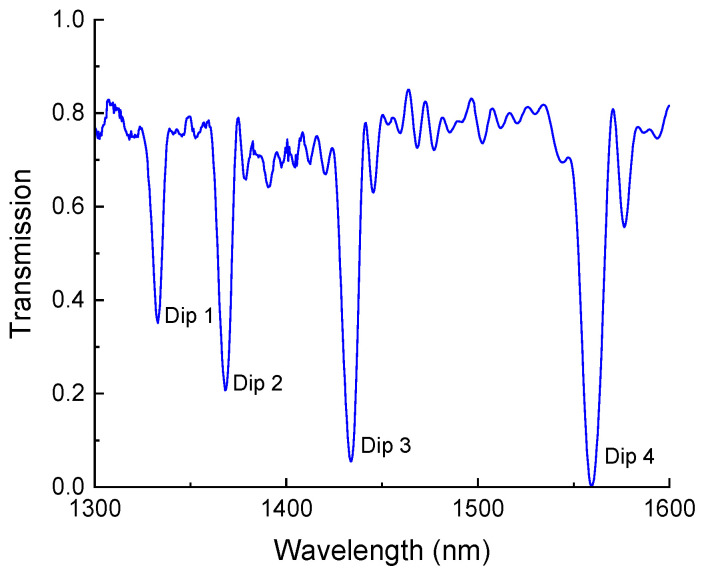
Transmission spectrum of LPFG in air with four cladding mode resonances.

We used long-period gratings in hydrogenated standard single-mode fiber (Corning SMF-28e) inscribed with a 244 nm UV laser using the direct point-by-point inscription method. The gratings had a period of 465 µm and a total length of 42 mm. A typical spectrum of the grating is shown in Figure 2. The center wavelengths of the attenuation bands of this LPFG when in air were 1333, 1368, 1434, and 1559 nm.

**Figure 3:**
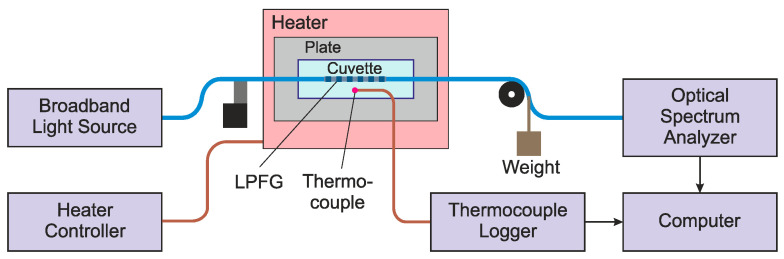
Scheme of experimental setup.

An experimental setup was developed to measure the spectral response of the LPFG. The setup consisted of a fiber optic broadband light source (EG&G Model OP507 1550, Boston, MA, USA), an Optical spectrum analyzer (Anritsu MS9740B, Atsugi, Japan), a long-period fiber grating, a cuvette, and a translation stage, as shown in Figure 3. To monitor the temperature variation, a K-type thermocouple and a Thermocouple Data Logger system (TC-08 from Pico Technology, Eaton Socon, UK) was used. The epoxy sample was poured into a custom-made sample holder developed with access points for the fiber to go through and was fixed on the hotplate. To strain the fiber, a weight was attached to the optical fiber or to the hardened resin sample to create a pulling force. To ensure better thermal homogeneity for measurements with the hardened resin sample, the sample was placed inside an aluminum tube with a rectangular profile (30 mm × 10 mm). The tube, with light insulation plugs at its ends, was laid directly on the hotplate and was covered with a thermal insulation blanket.

**Figure 4:**
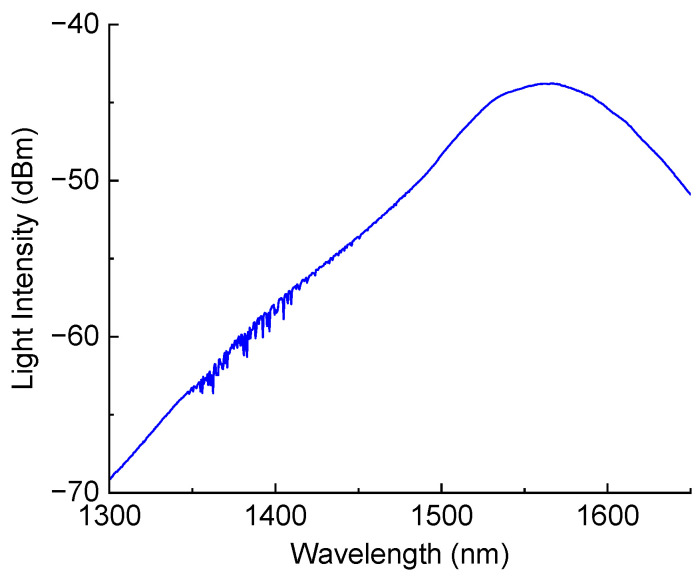
Spectrum of the light source.

EL2 Epoxy Laminating Resin and AT30 Slow Epoxy Hardener from EasyComposites (Rijen, The Netherlands) were mixed to prepare the resin. The spectrum of the light source is shown in Figure 4. It can be used to measure transmission spectra of LPFGs in the wavelength range 1250–1650 nm. Light from the broadband source was transmitted through the LPFG connected by standard fibers to the optical spectrum analyzer.

## 4. Sensitivity of LPFG in Air

**Figure 5:**
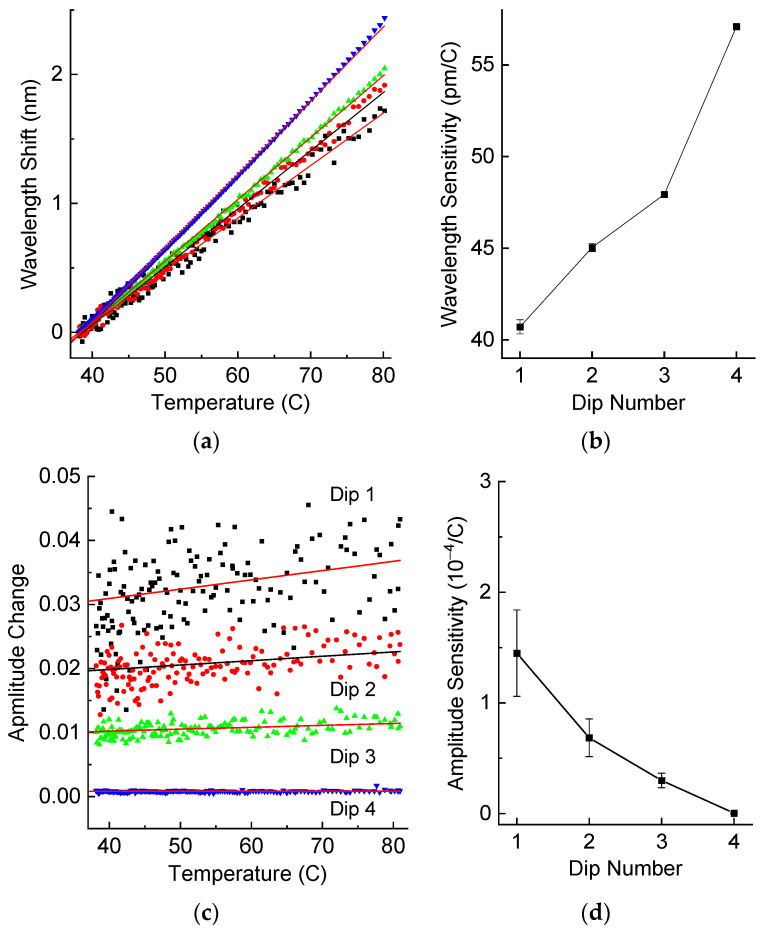
(**a**) Wavelength shifts and (**b**) wavelength sensitivity, (**c**) amplitude change and (**d**) amplitude sensitivity of resonance dips of the LPFG in air as a function of temperature.

It is well known that LPFGs are temperature sensitive. We measured the spectra of our LPFG in air with the temperature changing between room temperature and 80 °C. The results are presented in Figure 5, which shows the wavelength shift and the amplitude change for the four resonance dips. In order to find exact values of wavelength and amplitude, we fitted the tip of each dip with parabola. A total of 21 points around the peak value were taken into account for the fitting, which allowed us to reduce noise and increase measurement accuracy.

We can observe in Figure 5 that all the responses are quite linear. We introduced offsets of 0.01 units for successive dips at zero strain for better visual discrimination between shifts of different dips. Similar offsets are introduced in subsequent plots. The scattering of data points decreases with dip number. This is related to higher intensity of the superluminescent light source at longer wavelengths (see Figure 4), resulting in a higher signal to noise ratio. The peak intensity of the source is at 1550 nm. The difference between the light source intensities at wavelengths of the fourth and the first dips is about 130 times. The wavelength sensitivities for the four dips are not very different from each other and lie in the range of 40–57 pm/°C. The amplitude depends more strongly on temperature for the dips at shorter wavelength. The amplitude sensitivity of the fourth dip is practically zero.

**Figure 6:**
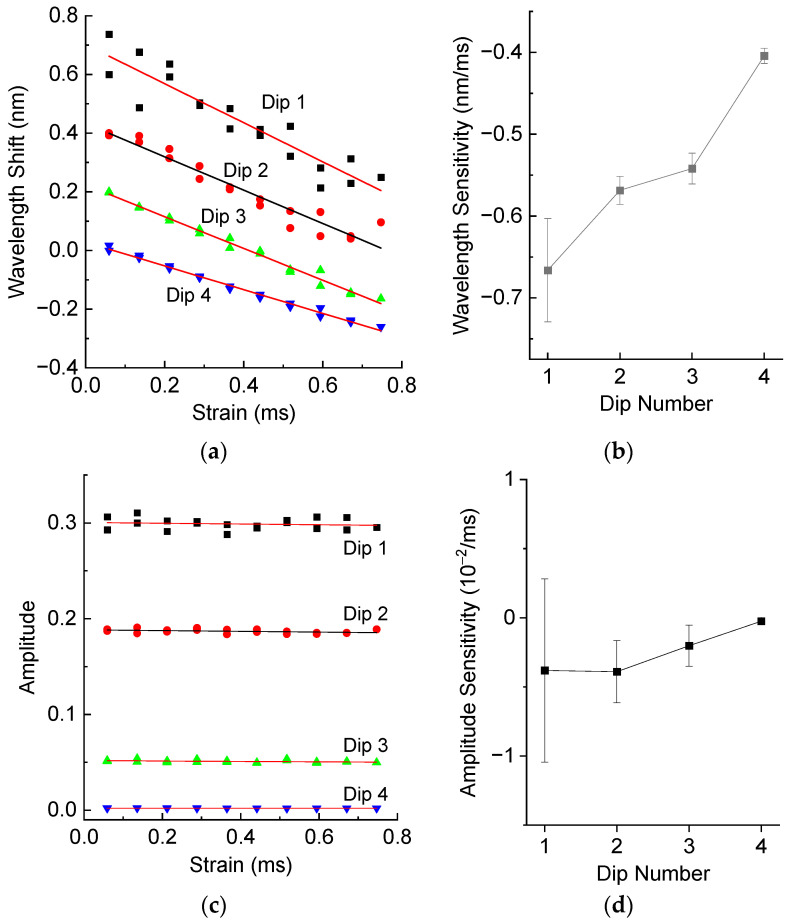
(**a**) Wavelength shifts and (**b**) wavelength sensitivity, (**c**) amplitude and (**d**) amplitude sensitivity of resonance dips of the LPFG in air as a function of strain.

The strain measurements were made by applying a series of weights attached to the fiber with the LPFG. The strain values were calculated knowing fiber dimensions and Young’s modulus of fused silica. Figure 6 shows wavelength shifts and wavelength sensitivity, amplitude and amplitude sensitivity of resonance dips of the LPFG as a function of strain. The strain sensitivities are all negative. The first dips demonstrate higher absolute wavelength sensitivity than the last. The amplitudes are almost independent of strain, with the sensitivity values close to zero within the error limits.

Let us describe the dependence of wavelength shifts and amplitude changes as linear functions of temperature and strain. We introduce a matrix M that relates these parameters:

(2) $$\left(\begin{array}{c} \Delta \lambda \\ \Delta P \end{array}\right)=M\left(\begin{array}{c} \Delta T\\ S_{f} \end{array}\right),\;M=\left(\begin{array}{cc} L_{T} & L_{Sf}\\ P_{T} & P_{Sf} \end{array}\right)$$

Here, ΔT is the temperature change and $S_{f}$ is the fiber strain. Δλ is the vector with four wavelength shifts and ΔP is the vector with four amplitude changes in the transmitted power at the dip centers:

(3) $$\Delta \lambda =\left[\begin{array}{c} \begin{array}{c} {\Delta \lambda }_{1}\\ {\Delta \lambda }_{2}\\ {\Delta \lambda }_{3} \end{array}\\ {\Delta \lambda }_{4} \end{array}\right],\Delta P=\left[\begin{array}{c} \begin{array}{c} {\Delta P}_{1}\\ {\Delta P}_{2}\\ {\Delta P}_{3} \end{array}\\ {\Delta P}_{4} \end{array}\right]$$

The matrix M in (2) is 2 × 2 literally but 8 × 2 practically and contains 16 elements. $L_{T}$ and $P_{T}$ are 4 × 1 matrices relating temperature change ΔT to wavelength shifts Δλ and amplitude changes ΔP. $L_{Sf}$ and $P_{Sf}$ are similar matrices describing the spectral response to fiber strain $S_{f}$. From the experimental results shown in Figure 5b,d and Figure 6b,d, we can find the elements of this matrix:

(4) $$L_{T}=\left[\begin{array}{c} \begin{array}{c} 40.71\\ 45.04\\ 47.94 \end{array}\\ 57.09 \end{array}\right]\frac{pm}{{}^{\circ}C},\;{\delta L}_{T}=\left[\begin{array}{c} \begin{array}{c} 0.39\\ 0.23\\ 0.12 \end{array}\\ 0.10 \end{array}\right]\frac{pm}{{}^{\circ}C},\;L_{Sf}=\left[\begin{array}{c} \begin{array}{c} -666.2\\ -568.6\\ -541.9 \end{array}\\ -404.2 \end{array}\right]\frac{pm}{m\varepsilon },\;{\delta L}_{Sf}=\left[\begin{array}{c} \begin{array}{c} 63\\ 17\\ 19 \end{array}\\ 9.4 \end{array}\right]\frac{pm}{m\varepsilon },$$

(5) $$P_{T}=\left[\begin{array}{c} \begin{array}{c} 1.45\\ 0.685\\ 0.299 \end{array}\\ 0.0034 \end{array}\right]\frac{10^{-4}}{{}^{\circ}C},{\delta P}_{T}=\left[\begin{array}{c} \begin{array}{c} 0.39\\ 0.17\\ 0.065 \end{array}\\ 0.0081 \end{array}\right]\frac{10^{-4}}{{}^{\circ}C},\;P_{Sf}=\left[\begin{array}{c} \begin{array}{c} -38.1\\ -39.0\\ -20.2 \end{array}\\ -2.44 \end{array}\right]\frac{10^{-4}}{m\varepsilon },{\delta P}_{Sf}=\left[\begin{array}{c} \begin{array}{c} 66.3\\ 22.6\\ 14.9 \end{array}\\ 1.09 \end{array}\right]\frac{10^{-4}}{m\varepsilon }$$

where ${\delta L}_{T}$, ${\delta P}_{T}$, ${\delta L}_{Sf}$, and ${\delta P}_{Sf}$ are the corresponding measurement errors.

To solve the inverse problem of finding temperature and strain from grating spectra, we need to calculate the matrix inverse of M. In our case, the matrix is not square, therefore, we can use the pseudo-inverse matrix, which is based on the least squares method. The data in the matrix have different dimensions, and the pseudo-inverse matrix would depend on the measurement units that are used to write the matrix. On the other hand, different parameters are measured with different errors. More accurate measurements should have larger weights when using the least squares method. To account for that, we introduce a diagonal matrix of weights W that is formed from the measurement errors combined from both the temperature and the strain measurements:

(6) $$W=\mathrm{diag}\left({\left({\left(∆T_{m}{\delta L}_{T}\right)}^{2}+{\left(∆S_{m}{\delta L}_{S}\right)}^{2}\right)}^{-1/2},\;{\left({\left(∆T_{m}{\delta P}_{T}\right)}^{2}+{\left(∆S_{m}{\delta P}_{Sf}\right)}^{2}\right)}^{-1/2}\right)$$

Here, $∆T_{m}$ and $∆S_{m}$ are the ranges of temperature and strain variation. In this case,

(7) $$∆T_{m}=55\;{}^{\circ}C,\;∆S_{m}=0.75\;m\varepsilon$$

We can obtain the following numerical values for the weight matrix from Figure 5b,d and Figure 6b,d:

(8) $$W=\mathrm{diag}{\left(\left[51.9,18.0,15.5,8.9\right]1/\mathrm{pm},\left[54.2,19.4,11.7,0.9\right]10^{4}\right)}^{-1}$$

Taking the weight matrix into account, we find the inverse relationship between wavelength shifts and amplitude changes with temperature and strain:

(9) $$\left(\begin{array}{c} \Delta T\\ S_{f} \end{array}\right)={\left[W\left(\begin{array}{cc} L_{T} & L_{Sf}\\ P_{T} & P_{Sf} \end{array}\right)\right]}^{+}W\left(\begin{array}{c} \Delta \lambda \\ \Delta P \end{array}\right)$$

where the cross denotes the pseudo-inverse of a matrix.

## 5. Sensitivity of LPFG in Liquid Resin

Due to excitation of cladding modes, LPFGs are sensitive to the external RI and their temperature and strain sensitivities may change inside different media. We measured the spectra of our LPFG in resin with changing external RI, temperature, and strain applied to the fiber.

**Figure 7:**
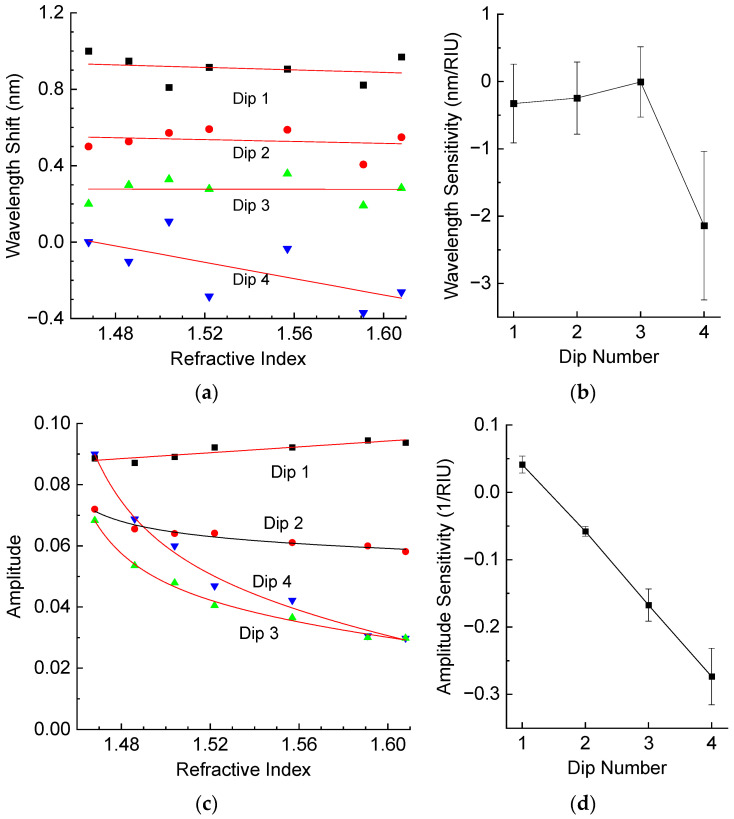
(**a**) Wavelength shifts and (**b**) wavelength sensitivity, (**c**) amplitude and (**d**) amplitude sensitivity at *n* = 1.53 of resonance dips of the LPFG as a function of external RI.

The RI of the external medium used is higher than that of silica, and we observe mainly amplitude change with changing RI. We used a set of liquids from Cargille with different RIs in the range 1.47–1.61 (at a wavelength of 1550 nm). The fiber was placed straight in a groove under slight tension. Then, the groove was filled with the RI liquid and a spectrum was measured. After each measurement, we wiped the groove and the fiber using alcohol. The measured results are shown in Figure 7, which demonstrates the wavelength shift and the amplitude change for four resonance dips. The wavelength shifts are quite small for all the dips. The amplitudes of Dips 1 and 2 have lower RI sensitivity, and Dip 1 has positive sensitivity compared to the other dips. In general, the amplitude dependence on RI is nonlinear with increasing gradient close to the RI of silica (1.444) [9]. In terms of our application, we are interested in RIs close to the RI of the resin, which is, in our case, about 1.53 for the liquid resin and 1.56 for the hardened resin. We can linearize these variations at this point, and the resulting sensitivity is shown in Figure 7d. The highest sensitivity is observed for Dip 4, and it is equal to –2.7 1/RIU.

**Figure 8:**
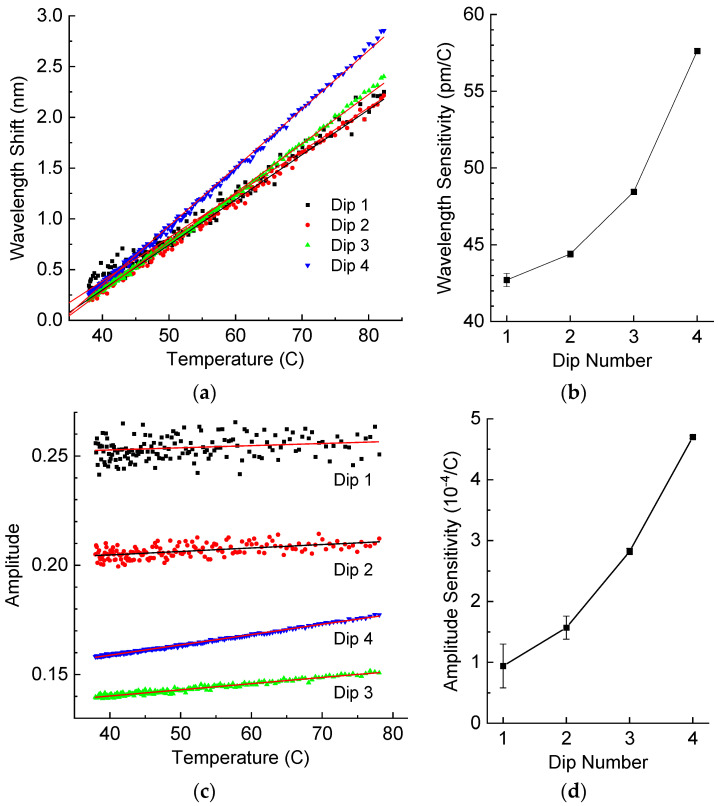
(**a**) Wavelength shifts and (**b**) wavelength sensitivity, (**c**) amplitude and (**d**) amplitude sensitivity of resonance dips of the LPFG in liquid resin as a function of temperature.

The temperature response of LPFG was measured by slow cooling of the LPFG placed in uncured resin from a temperature of 82 °C to room temperature (Figure 8). By comparing with the results in Figure 6 obtained in air, we can see that the wavelength shifts are quite similar in the presence of resin and lie in the range 40–60 pm/°C. On the other hand, the presence of resin changes the dependence of dips’ amplitudes on temperature, which can be accounted to decreasing RI due to the thermo-optic effect in the resin.

**Figure 9:**
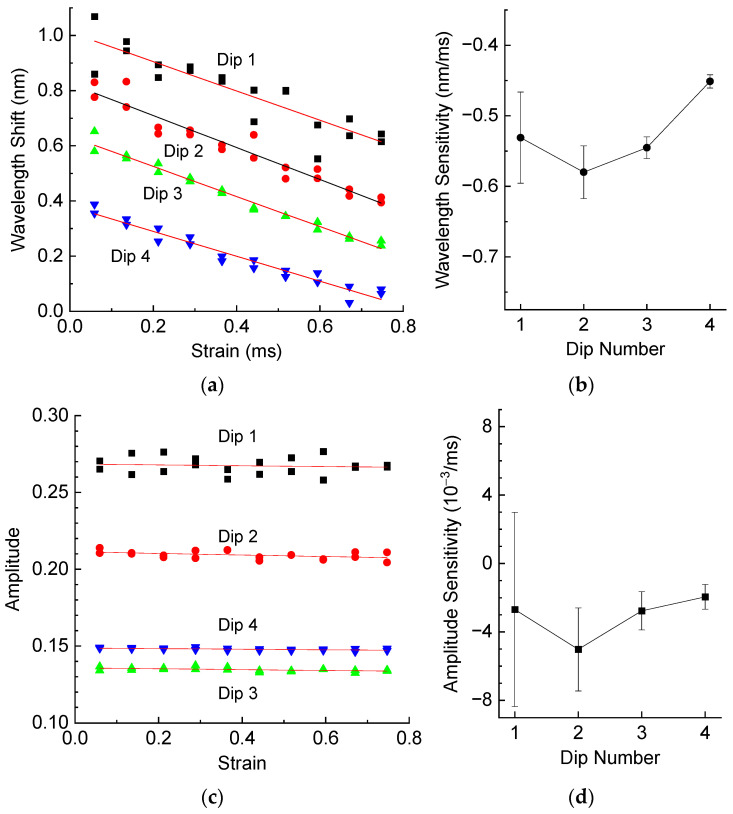
(**a**) Wavelength shifts and (**b**) wavelength sensitivity, (**c**) amplitude and (**d**) amplitude sensitivity of resonance dips of the LPFG in liquid resin as a function of strain.

The strain measurements were made in a similar manner to those of fiber in air except for submersing the fiber with LPFG into liquid resin. Figure 9 shows wavelength shifts and wavelength sensitivity, amplitude and amplitude sensitivity of resonance dips of the LPFG as a function of strain. The wavelength strain sensitivities are all negative and about –0.5 nm/mε. The amplitudes are almost independent of strain, with the sensitivity values close to zero within the error limits.

The dependence of wavelength shifts and amplitude changes as functions of temperature, strain, and external RI can be described in a similar manner to previously using the following matrix:

(10) $$\left(\begin{array}{c} \Delta \lambda \\ \Delta P \end{array}\right)=\left(\begin{array}{ccc} L_{T} & L_{Sf} & L_{Ne}\\ P_{T} & P_{Sf} & P_{Ne} \end{array}\right)\left(\begin{array}{c} \Delta T\\ S_{f}\\ {\Delta n}_{e} \end{array}\right)$$

The wavelength shifts ($L_{T}$) and amplitude changes ($P_{T}$) of resonance dips with increasing fiber temperature are induced by two factors: the thermal effect in the fiber and the thermo-optic effect changing the external RI. Therefore, if we introduce the thermo-optic coefficient $N_{Tl}$ of the external liquid, then the matrix relation can be written in the following form:

(11) $$\left(\begin{array}{c} \Delta \lambda \\ \Delta P \end{array}\right)=\left(\begin{array}{ccc} L_{Ta}+\Lambda_{Ne}N_{Tl} & L_{Sf} & \Lambda_{Ne}\\ P_{Ta}+\Pi_{Ne}N_{Tl} & P_{Sf} & \Pi_{Ne} \end{array}\right)\left(\begin{array}{c} \Delta T\\ S_{f}\\ {\Delta n}_{e} \end{array}\right)$$

where $\Lambda_{Ne}$ and $\Pi_{Ne}$ describe the spectral responses to changing external RI, and $L_{Ta}$ and $P_{Ta}$ are the matrix elements for the LPFG in air. The RI value in the third row of the parameter vector is the value taken for room temperature ${\Delta n}_{e}={\Delta n}_{e}(25C)$. The change in RI with temperature due to the thermo-optic effect is accounted by $N_{Tl}$ in the elements of the matrix. The inverse relationship for the case of variable external RI is described by the pseudo-inverse matrix taking the weights into account:

(12) $$\left(\begin{array}{c} \Delta T\\ S_{f}\\ {\Delta n}_{e} \end{array}\right)={\left[W\left(\begin{array}{ccc} L_{T} & L_{Sf} & L_{Ne}\\ P_{T} & P_{Sf} & P_{Ne} \end{array}\right)\right]}^{+}W\left(\begin{array}{c} \Delta \lambda \\ \Delta P \end{array}\right)$$

From experimental data shown in Figure 9, we can write the elements of the matrix and the associated measurement errors:

(13) $$L_{Ne}=\left[\begin{array}{c} \begin{array}{c} -327\\ -246\\ -5 \end{array}\\ -2140 \end{array}\right]\frac{pm}{RIU},{\delta L}_{Ne}=\left[\begin{array}{c} \begin{array}{c} 584\\ 535\\ 522 \end{array}\\ 1103 \end{array}\right]\frac{pm}{RIU},\;$$

$$P_{Ne}=\left[\begin{array}{c} \begin{array}{c} 412\\ -578\\ -1673 \end{array}\\ -2735 \end{array}\right]\frac{10^{-4}}{RIU},\;{\delta P}_{Ne}=\left[\begin{array}{c} \begin{array}{c} 126\\ 71\\ 239 \end{array}\\ 419 \end{array}\right]\frac{10^{-4}}{RIU}$$

(14) $$L_{T}=\left[\begin{array}{c} \begin{array}{c} 42.70\\ 44.39\\ 48.45 \end{array}\\ 57.63 \end{array}\right]\frac{pm}{{}^{\circ}C},\;{\delta L}_{T}=\left[\begin{array}{c} \begin{array}{c} 0.43\\ 0.21\\ 0.14 \end{array}\\ 0.17 \end{array}\right]\frac{pm}{{}^{\circ}C},\;L_{Sf}=\left[\begin{array}{c} \begin{array}{c} -530\\ -579\\ -545 \end{array}\\ -451 \end{array}\right]\frac{pm}{m\varepsilon },\;{\delta L}_{Sf}=\left[\begin{array}{c} \begin{array}{c} 65\\ 37\\ 15 \end{array}\\ 9 \end{array}\right]\frac{pm}{m\varepsilon }$$

(15) $$P_{T}=\left[\begin{array}{c} \begin{array}{c} -531\\ -580\\ -545 \end{array}\\ -451 \end{array}\right]\frac{10^{-4}}{{}^{\circ}C},{\delta P}_{T}=\left[\begin{array}{c} \begin{array}{c} 65\\ 37\\ 15 \end{array}\\ 9 \end{array}\right]\frac{10^{-4}}{{}^{\circ}C},P_{Sf}=\left[\begin{array}{c} \begin{array}{c} -27\\ -50\\ -28 \end{array}\\ -19 \end{array}\right]\frac{10^{-4}}{m\varepsilon },{\delta P}_{Sf}=\left[\begin{array}{c} \begin{array}{c} 57\\ 24\\ 11 \end{array}\\ 7 \end{array}\right]\frac{10^{-4}}{m\varepsilon }$$

From the measurement errors and the ranges of RI, temperature, and strain variation, which are $∆n=0.1,∆T_{m}=55\;{}^{\circ}C,\;∆S_{m}=0.75\;m\varepsilon$ in this case, we obtain the following numerical values for the weight matrix:

(16) $$W=\mathrm{diag}{\left(\left[79.5,61.5,54.0,111.0\right]1/\mathrm{pm},\left[48.7,22.2,25.5,42.3\right]10^{4}\right)}^{-1}$$

## 6. Sensitivity in Hardened Resin

The next step in the analysis of interaction between LPFG and resin is the study of hardened resin. The hardened resin holds the fiber and elongates it when strained or heated. Therefore, the behavior of LPFG in hardened resin is different from that in liquid resin. We measured the spectra of the LPFG in hardened resin with temperature and strain applied to the resin sample. The sample was prepared in a flexible cuvette with inner sizes 85 × 23 mm and filled with resin with a thickness of 3.1 mm. The LPFG was placed in the middle of the cuvette. The resin was cured for 24 h at room temperature followed by 6 h at 60 °C. Then, the sample was removed from the cuvette and its sides were cut off, so that the width in the region of the grating was 7.5 mm and the sample cross-section was 7.5 × 3.1 mm. For the temperature measurements, the sample was placed on a hotplate. For the strain measurements, one end of the sample was fixed, and the other end was attached to the load. The strain was calculated from the load using the sample cross-section and tensile strength of the hardened resin (69.5 MPa).

**Figure 10:**
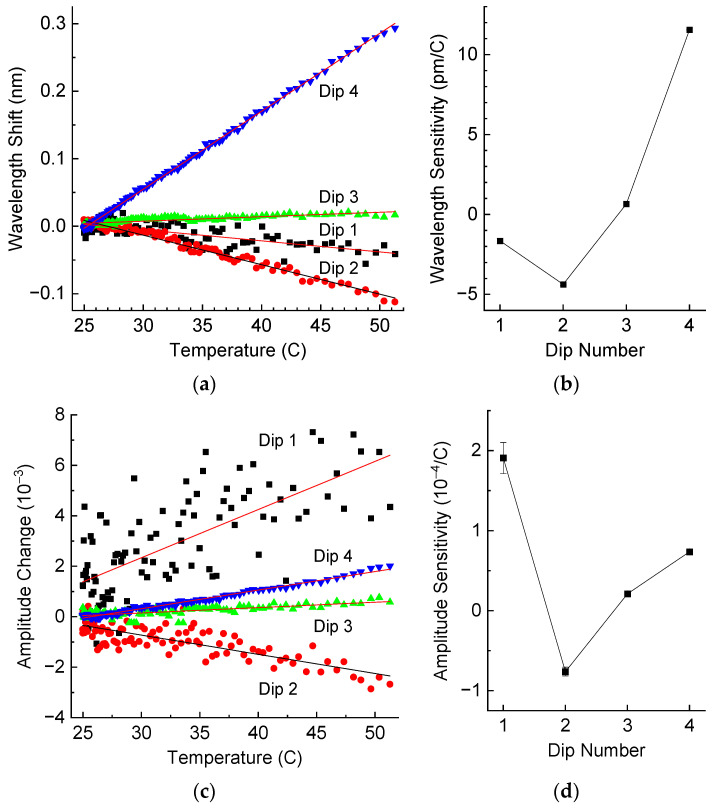
(**a**) Wavelength shifts and (**b**) wavelength sensitivity, (**c**) amplitude change and (**d**) amplitude sensitivity of resonance dips of the LPFG in hardened resin as a function of temperature.

We measured the spectra of LPFG inside the resin sample with the temperature changing between room temperature and 52 °C. The results are presented in Figure 10, which shows the wavelength shifts and amplitude changes for the four resonance dips. We can observe from Figure 10 that the dependencies are quite linear. The sensitivities have different signs: the wavelength sensitivities are positive for Dips 3 and 4 and negative for Dips 1 and 2; the amplitude sensitivities are positive for Dips 1, 3, and 4 and negative for Dip 2. This is very different from what we observed for liquid resin. The strongest wavelength shift is about 11 pm/°C for Dip 4 with the least error. However, this is much lower than that for the LPFG in air or liquid resin.

**Figure 11:**
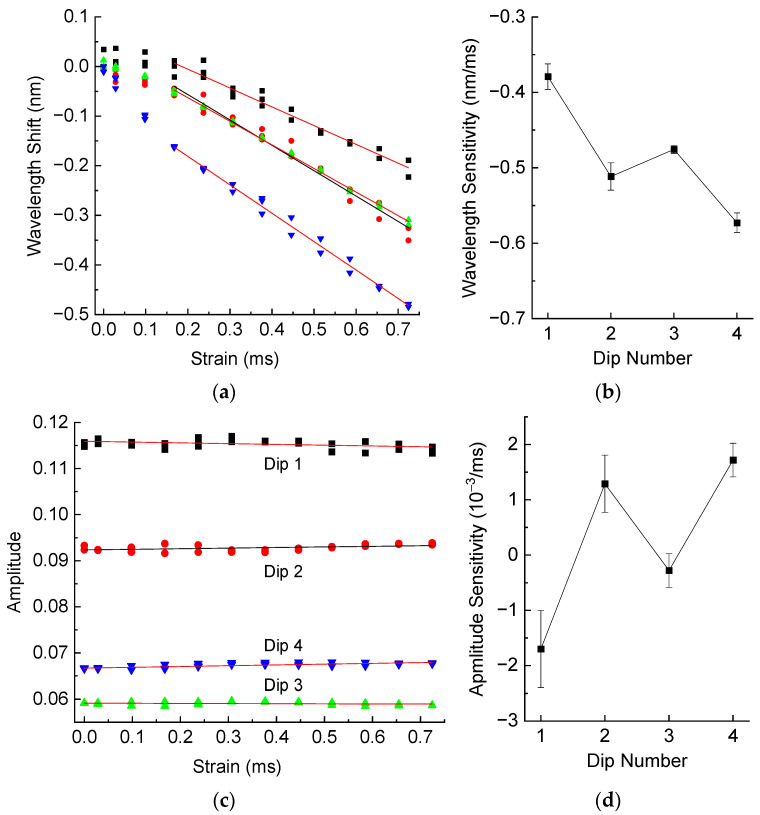
(**a**) Wavelength shifts and (**b**) wavelength sensitivity, (**c**) amplitude and (**d**) amplitude sensitivity of resonance dips of the LPFG in hardened resin as a function of strain.

The results of strain measurements for the LPFG inside hardened resin are presented in Figure 11. The wavelength shifts are nonlinear functions of strain for strains up to 0.15 mε, which is, probably, related to some initial straightening of the resin sample for small loads. If we neglect the data for strains below 0.15 mε, the wavelength strain sensitivities are all negative and about –0.5 nm/mε, as is shown in Figure 11. This is similar to the case of liquid resin. The amplitudes have low dependence on strain, with the sensitivity values not far from zero taking the error limits into account.

In cases where we have strain in both the fiber and the resin, the matrix relating temperature, fiber strain ($S_{f}$), and resin strain ($S_{e}$) with spectral characteristics has the form:

(17) $$\left(\begin{array}{c} \Delta \lambda \\ \Delta P \end{array}\right)=\left(\begin{array}{ccc} L_{T} & L_{Sf} & L_{Se}\\ P_{T} & P_{Sf} & P_{Se} \end{array}\right)\left(\begin{array}{c} \Delta T\\ S_{f}\\ S_{e} \end{array}\right)$$

Since the fiber cannot move independently of resin, the fiber strain $S_{f}=S_{e}$. The matrix relationship can be rewritten as follows:

(18) $$\left(\begin{array}{c} \Delta \lambda \\ \Delta P \end{array}\right)=\left(\begin{array}{cc} L_{T} & L_{Sh}\\ P_{T} & P_{Sh} \end{array}\right)\left(\begin{array}{c} \Delta T\\ S_{e} \end{array}\right)=\left(\begin{array}{cc} L_{Ta}+\Lambda_{Ne}N_{Th} & L_{Sf}+L_{Se}\\ P_{Ta}+\Pi_{Ne}N_{Th} & P_{Sf}+P_{Se} \end{array}\right)\left(\begin{array}{c} \Delta T\\ S_{e} \end{array}\right)$$

where $N_{Th}$ is the thermo-optic coefficient of the hardened resin.

The inverse formula for the case of hardened resin allows one to find temperature change and external strain using the pseudo-inverse matrix:

(19) $$\left(\begin{array}{c} \Delta T\\ S_{e} \end{array}\right)={\left[W\left(\begin{array}{cc} L_{T} & L_{Sh}\\ P_{T} & P_{Sh} \end{array}\right)\right]}^{+}W\left(\begin{array}{c} \Delta \lambda \\ \Delta P \end{array}\right)$$

From experimental data shown in Figure 10 and Figure 11, we can write the elements of the matrix and the measurement errors:

(20) $$L_{T}=\left[\begin{array}{c} \begin{array}{c} -1.65\\ -4.37\\ 0.66 \end{array}\\ 11.55 \end{array}\right]\frac{pm}{{}^{\circ}C},\;{\delta L}_{T}=\left[\begin{array}{c} \begin{array}{c} 0.15\\ 0.07\\ 0.04 \end{array}\\ 0.03 \end{array}\right]\frac{pm}{{}^{\circ}C},\;L_{Se}=\left[\begin{array}{c} \begin{array}{c} -379\\ -511\\ -476 \end{array}\\ -573 \end{array}\right]\frac{pm}{m\varepsilon },\;{\delta L}_{Se}=\left[\begin{array}{c} \begin{array}{c} 16\\ 18\\ 5 \end{array}\\ 13 \end{array}\right]\frac{pm}{m\varepsilon }$$

(21) $$P_{T}=\left[\begin{array}{c} \begin{array}{c} 1.908\\ -0.762\\ 0.211 \end{array}\\ 0.736 \end{array}\right]\frac{10^{-4}}{{}^{\circ}C},{\delta P}_{T}=\left[\begin{array}{c} \begin{array}{c} 0.194\\ 0.055\\ 0.019 \end{array}\\ 0.009 \end{array}\right]\frac{10^{-4}}{{}^{\circ}C},$$

$$P_{Se}=\left[\begin{array}{c} \begin{array}{c} -17.0\\ 12.9\\ -2.8 \end{array}\\ 17.2 \end{array}\right]\frac{10^{-4}}{m\varepsilon },{\delta P}_{Se}=\left[\begin{array}{c} \begin{array}{c} 7.0\\ 5.2\\ 3.1 \end{array}\\ 3.0 \end{array}\right]\frac{10^{-4}}{m\varepsilon }$$

The ranges of temperature and strain variation in this experiment were $∆T_{m}=25\;{}^{\circ}C,\;∆S_{m}=0.72\;ms$. The weight matrix has the following elements:

(22) $$W=\mathrm{diag}{\left(\left[12.7\;13.2\;3.8\;9.3\right]1/pm,\left[6.0\;4.0\;2.3\;2.2\right]10^{4}\right)}^{-1}$$

We should note that there is some strain of fiber caused by shrinking of the resin; however, while this additional strain shifts the dips and changes dip amplitudes during the curing process, it is constant after full curing and it does not depend on temperature and external strain. We noticed that curing lowers the dips of the LPFG. At the first stage of curing at constant temperature, wavelength shifts are small, while dips’ amplitudes are affected, because additional strain is accumulated during high-temperature curing. We noted that the amplitudes and wavelengths are not single-valued functions of temperature; there is a memory effect even for hardened resin and hysteresis-like behavior during heating–cooling cycles.

## 7. Calculation

**Figure 12:**
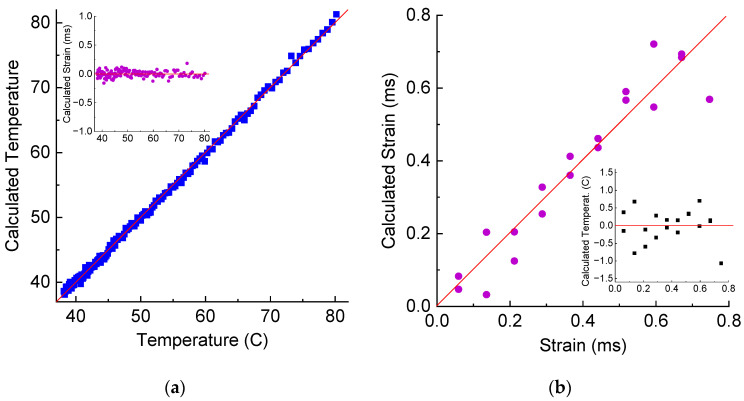
Calculation of strain and temperature from wavelength and amplitudes of LPFG’s dips using pseudo-inverse matrix during (**a**) heating and (**b**) straining.

Now we have obtained all the matrices that describe spectral changes in the cases of the LPFG in air, liquid, and hardened resin. From Equation (9), we can find the pseudo-inverse matrix for the LPFG in air:

(23) $$\left(\begin{array}{c} \Delta T\\ S_{f} \end{array}\right)=10^{-3}\left(\begin{array}{c} -2.954\;-13.06\;-11.70\;39.81\;-0.229\;-2.364\;-3.433\;-74.812\\ -0.378\;-1.823\;-1.789\;3.218\;-0.028\;-0.282\;-0.408\;-8.814 \end{array}\right)\left(\begin{array}{c} \Delta \lambda \\ \Delta P \end{array}\right)$$

where wavelength is measured in pm, amplitude in $10^{-4}$, temperature in °C, and strain in mε. We can use this matrix to decouple temperature and strain from the original spectral measurements in air. The calculation results are shown in Figure 12. As can be seen from the figure, we successfully reconstructed the two parameters with standard deviations of about 0.46 °C in temperature and 0.056 mε in strain.

For the case of LPFG in a liquid, we can find the following matrix relationship including the RI of the liquid:

(24) $$\left(\begin{array}{c} \Delta T\\ S_{f}\\ {\Delta n}_{e} \end{array}\right)=10^{-3}\left(\begin{array}{c} -5.829\;-20.940\;16.475\;26.723\;-5.565\;-109.209\;-15.486\;13.385\\ -0.819\;-2.363\;0.633\;2.117\;-0.534\;-9.427\;-1.149\;1.270\\ 0.0012\;-0.0011\;0.0335\;0.0027\;0.0166\;-0.183\;-0.281\;-0.154 \end{array}\right)\left(\begin{array}{c} \Delta \lambda \\ \Delta P \end{array}\right)$$

**Figure 13:**
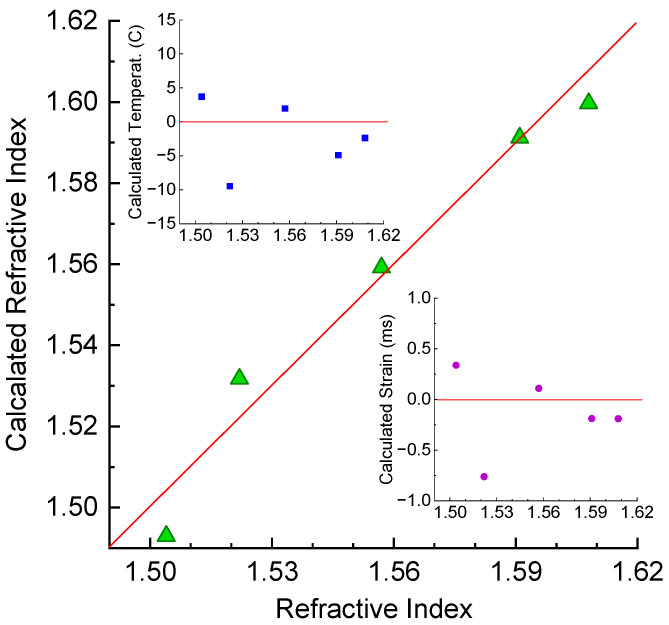
Calculation of RI from wavelength and amplitudes of LPFG’s dips using pseudo-inverse matrix in liquid resin.

The results the of calculation of RI from the wavelength shifts and amplitude changes are presented in Figure 13. The standard deviations for RI, temperature, and strain are about 0.008, 5.3 °C, and 0.4 mε, respectively. The error is one order of magnitude higher than in the case of air and two independent parameters. Lower accuracy is also related to having fewer experimental points in the measurement of RI.

For the case of LPFG in hardened resin, we obtain the following matrix:

(25) $$\left(\begin{array}{c} \Delta T\\ S_{e} \end{array}\right)=10^{-3}\left(\begin{array}{c} -11.61\;-22.10\;-45.10\;66.53\;23.16\;-27.69\;24.07\;101.98\\ -0.1522\;-0.2181\;-1.6579\;-0.061\;0.0689\;-0.0638\;0.063\;0.5404 \end{array}\right)\left(\begin{array}{c} \Delta \lambda \\ \Delta P \end{array}\right)$$

**Figure 14:**
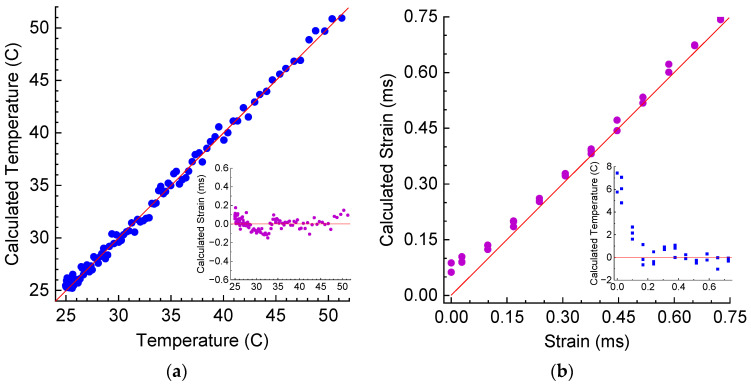
Calculation of temperature and strain from wavelength and amplitudes of LPFG’s dips using pseudo-inverse matrix in hardened resin during (**a**) heating and (**b**) straining.

The calculated temperature and strain from the wavelength shifts and amplitude changes are presented in Figure 14. The standard deviations for temperature and strain are 0.5 °C and 0.064 mε, which are similar to those for the LPFG in air. For variable strain, we observe some deviation of calculated points both for temperature and strain from real values for small strains below 0.15. This is related to the nonlinearity of measured data for small strains, as noted above. Above these values the standard deviations are 0.58 °C and 0.019 mε.

In Table 1, we compare various multiparameter sensors based on LPFGs. As can be noted, our sensing scheme demonstrates characteristics similar to previous designs. At the same time, it provides RI measurements for epoxy resins having RIs above the refractive index of silica (*n* = 1.444).

In terms of application in wavelength multiplexed quasi-distributed fiber-optic sensor systems, LPFGs are difficult to use because they have much broader spectra compared to fiber Bragg gratings. In our case, it is even more difficult, since we use information about four different dips covering a large spectral range.

## 8. Conclusions

We have demonstrated that it is possible to decouple the effects of temperature, strain, and RI in long-period fiber grating used for epoxy resin cure monitoring. We have analyzed spectral wavelength shifts and dip amplitudes of four cladding mode notches, which provided us with eight partially independent parameters.

We have observed that wavelength shifts induced by temperature change are independent of the presence of liquid resin and are around 40–60 pm/°C. The amplitude change in this case depends on the presence of resin and is a result of decreasing RI due to the thermo-optic effect in the resin. In hardened resin, the amplitudes have low dependence on strain. The wavelength sensitivities to temperature have different signs for different modes, which is very different from the cases of air and liquid resin with strong positive wavelength shifts. This can be explained by elongation of the LPFG together with the hardened resin sample. The strongest amplitude change for the range of stimuli considered here was induced by changing the RI of the external medium.

From wavelength shifts and changes in dip amplitudes, we have been able to reconstruct temperature, strain, and RI for gratings in air, liquid, and hardened resins. The three values were found by employing multiplication of a weighted pseudo-inverse matrix, assuming linear dependences between the spectral and external parameters. The typical standard deviations for the reconstructed temperature, strain, and RI are 0.5 °C, 0.06 mε, and 0.008 RIU or 2%, 8%, and 13% of the full scale ranges. In order to improve the reconstruction of the measurands, we would explore using a light source with a more uniform power output over the wavelength range of interest and increasing the number of RI values at which measurements were taken.

We have proposed a model describing the influence of fiber parameters and external RI, temperature, and strain on the spectral behavior of long-period fiber gratings in epoxy resins during hardening. The results obtained can be applied for multiparameter cure process monitoring of epoxy resins by using long-period fiber gratings. The potential field of application of such sensors is the manufacturing of large composite structures, in particular, the manufacturing of wind turbine blades with sizes above 100 m. The sensors can be placed between the fiberglass mats at different locations before the infusion process and then used for monitoring of resin flow, changing refractive index (indicating the degree of resin curing), temperature, and strain. The LPFG length is usually a few centimetres and can be considered as having a homogeneous environment along its length in structures with sizes of tens of meters.

## Figures and Tables

**Table 1:** Comparison of various multiparameter sensors based on LPFGs.

No	Sensor Type	Measurand	Accuracy/Sensitivity	Reference
1	LPFG	Strain, Temperature, RI	33 με, 0.66 C, 0.002 RIU (1.4 < *n* < 1.43)	[19]
2	LPFG	Strain, Temperature	58 με, 1 °C	[13]
3	Phase-shifted LPFG	Strain, Temperature, RI, pH	0.1225 nm/μϵ, 0.2128 nm/°C, 718 nm/RIU, –1.0166 nm/pH	[20]
4	Ultra-long LPFG	Curvature, Temperature	69.43 dB∙m, 62.94 pm/°C	[21]
5	Phase-shifted LPFG	Torsion, Temperature	0.07 nm/(rad/m), 0.05 nm/°C	[22]
6	LPFG	Strain, Temperature, RI	60 με, 0.5 °C, 0.008 RIU (1.46 < *n* < 1.61)	This work

## Data Availability

The original contributions presented in the study are included in the article, further inquiries can be directed to the corresponding author.
